# A meta‐analysis of the effect of probiotic administration on age‐related sarcopenia

**DOI:** 10.1002/fsn3.3515

**Published:** 2023-08-09

**Authors:** Nafiseh Shokri‐Mashhadi, Fatemeh Navab, Shakila Ansari, Mohammad Hossein Rouhani, Zahra Hajhashemy, Sahar Saraf‐Bank

**Affiliations:** ^1^ Department of Clinical Nutrition, School of Nutrition and Food Science, Nutrition and Food Security Research Center Isfahan University of Medical Sciences Isfahan Iran; ^2^ Department of Community Nutrition, School of Nutrition and Food Science, Nutrition and Food Security Research Center Isfahan University of Medical Sciences Isfahan Iran

**Keywords:** muscle‐function, muscle‐strength, probiotics, sarcopenia

## Abstract

Global increase in the prevalence of age‐related diseases, such as sarcopenia, highlights the need of recognizing agents that improve muscle health; however, the evidence synthesis on the impact of probiotic administration on sarcopenia is scarce. To summarize and evaluate findings regarding the effect of supplementation with probiotics on sarcopenia, this meta‐analysis was conducted. Using databases, including PubMed, SCOPUS, ISI–Web of Science, and Cochrane Library, interventional studies were included if they investigate the effect of probiotic administration on at least one of the components of sarcopenia up to 6 October 2022. Risk of bias evaluation was conducted using the Cochrane quality assessment tool. The random‐effects model which takes between‐study variations into account was used to obtain the overall effect sizes. The STATA version 14.0 was used for statistical analyses. Overall, 17 studies were included. There was high certainty of evidence that probiotic supplementation has a beneficial effect on muscle mass (kg) (WMD: 0.55, 95% CI: 0.05, 1.05; *I* ^2^: 0.0%, *p* = .995), and muscle function (WMD: 0.13, 95% CI: 0.03, 0.23; *I* ^2^: 65.6%, *p* = .05). Moreover, administration of probiotics for more than 12 weeks significantly increased muscle strength (WMD: 1.16, 95% CI: 0.88, 1.44; *I* ^2^: 0.0%, *p* = .77). However, probiotic supplementation had no effect on anthropometric indices, including body mass index. Probiotic supplementation could improve muscle mass and muscle function in adults more than 55 years old. The beneficial effect of probiotics on muscle strength could appear after 12 weeks of supplementation.

## Graphical abstract

The global increase in the prevalence of age‐related diseases, such as sarcopenia, highlights the need of recognizing agents that improve muscle health; however, the evidence synthesis on the impact of probiotic administration on sarcopenia is scarce. To summarize and evaluate findings regarding the effect of supplementation with probiotics on sarcopenia, we conducted a meta‐analysis.

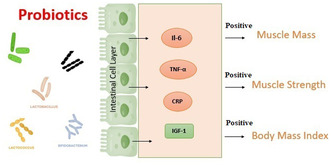

## 1 INTRODUCTION

The global elderly population is rapidly growing. Consequently, there would be a notable increase in the prevalence of age‐related diseases, namely, sarcopenia, and frailty (Partridge et al., 2018). The percentage of people over 60 years will approximately duplicate from 12% to 22% between 2015 and 2050 (Dhillon & Hasni, 2017; Partridge et al., 2018). According to the last definitions by EWGSOP, sarcopenia diagnosis is identified with low muscle strength, and it is considered severe if low muscle strength, low muscle mass, and low physical performance are detected (Cruz‐Jentoft et al., 2019). Poor muscle strength is associated with poor quality of life, risk of falls, fractures, and higher healthcare costs (Woo, 2017). Despite abundant efforts in ameliorating this disorder, current therapeutic approaches are not often associated with significant results (Iolascon et al., 2018). In this respect, a major challenge would be to recognize the factors protecting muscle health across the lifespan.

Epidemiologic observations indicated that changes in gut microbiota structure across the life course lead to distinctive microbiota composition and function (Lakshminarayanan et al., 2014; Ticinesi et al., 2017; Tiihonen et al., 2010). The reduction of microbiota bio‐diversities is associated with metabolic changes, physiologic dysregulation, and markers of inflammation that result in age‐related adverse health outcomes (Ticinesi et al., 2019). For this reason, researchers have hypothesized that gut microbiota composition may have a great relationship with age‐related modifications in skeletal muscle mass and function (Ticinesi et al., 2019). Recently, experimental studies revealed that changes in the gut ecosystem by using probiotics could affect the systemic inflammatory status and muscle function of aged animal models (Chen et al., 2016; Ni et al., 2019; Siddharth et al., 2017; Ticinesi et al., 2019). However, the clinical outcomes of probiotic administration in human trials are inconsistent (Aoyagi et al., 2019; Buigues et al., 2016; Picca et al., 2019; Sakata et al., 2019).

The main aim of this systematic review and meta‐analysis was to distinguish the impact of probiotic supplements on age‐related sarcopenic components. Moreover, the effect of probiotic administration on anthropometric indices in the adult population was assessed.

## 2 METHODS AND MATERIALS

### 2.1 Literature research and data sources

Current systematic review was carried out in compliance with the PRISMA statement (Moher et al., 2011). Two investigators (N. Sh‐M and F. Navab) independently conducted an electronic literature search using some reliable databases, including PubMed, SCOPUS, ISI–Web of Science, and Cochrane Library without any restrictions on language or data in order to identify the effect of probiotics on sarcopenia components when compared to standard care or placebo in elderly population (From inception to Oct 2022). The electronic search strategy was done using the following Medical Subject Headings (MESH) and non‐MESH keywords: (Sarcopenia OR “Muscle strength” OR “Hand strength” OR “Physical performance” OR “Muscle function*” OR Sarcopenia OR Frailty OR “Walking Speed” OR “Gait speed” OR “Grip strength*” OR “Hand grip*” OR “lean body mass” OR “Percentage of body fat” OR “Knee extension strength*”) AND (Probiotic* OR “Escherichia coli” OR Microbiota* OR Bifidobacterium OR Lactobacillus OR Saccharomyces OR kefir OR Yogurt) (Supplementary File 1). To expand the research, the impact of probiotics on anthropometric and body composition was assessed separately (“Quetelet Index” OR “Body Mass Index” OR “Body weight” OR “calf circumference” OR “Waist circumference*” OR “Body composition” OR BMI OR “skin‐fold thickness” OR “fat free mass” OR “body mass”) when compared to standard care or placebo in elderly population. The references pointed out in the retrieved articles were also searched manually.

### 2.2 Inclusion criteria

Through abstract reading, all studies with the following criteria were eligible to be included in the present review: (1) were interventional studies; (2) compared the effect of administration of probiotics alone as a supplementation versus nonprobiotics on at least one of the components of sarcopenia (muscle strength, muscle mass, muscle function) in adults (mean/median age of 55 years and older). Investigations were not included if they: (1) were duplicated publications; (2) were not conducted on adults (≥55); (3) were observational studies; (4) were done on animal models or in vivo studies; (5) were review or protocol articles; (6) were classified as gray literatures, for instance, conference abstracts, government reports, and theses. To define the impact of probiotics on body composition, all interventional studies that investigate the effect of supplementation with probiotics on at least one of the anthropometric measurements were considered.

### 2.3 Exclusion criteria

Studies were eligible to be excluded in the present review if: (1) the intervention included whole food or component of foods (such as dairy products) and did not report the dosage of probiotics; (2) reported the effect of multiple nutrients along with probiotics supplementation (multi supplement); (3) The study was conducted on adults (<55 years); (4) did not report the predefined endpoints as mean (±SD) for sarcopenia components (Supplementary File 2). After abstract reading, all studies, which assessed the effect of probiotics administration alone versus non‐probiotics in adults (>55 years), were included in this review. Additional outcomes were the data related to the effects of probiotics on anthropometric and body composition in elderly adults which were searched separately with predefined keywords, and results of the analysis are reported.

The following information was extracted: author's name, publication year, participants' characteristics (sample size, gender, and age), health status, intervention (type of compounds, dose, and duration), and main outcomes, including muscle mass, muscle strength, and muscle function. The data were extracted independently by two investigators (F.N and Z.H). We also contacted the corresponding author to obtain the data when necessary (N.Sh‐M).

### 2.4 Assessment of bias

Risk of bias assessment: In the current meta‐analysis, the Cochrane quality assessment tool was used to examine the risk of bias for each study included (Higgins et al., 2019). This tool consists of seven domains including reporting bias, detection bias, random sequence generation, allocation concealment, performance bias, attrition bias, and other sources of bias. Each domain was assigned a “high risk” score if there was a methodological flaw that could have affected the findings, a “low risk” score if the domain was not defective, and an “unclear risk score” if no information was available to determine the impact. The overall risk of bias for an RCT was considered: (1) Low; for studies that all their domains obtained “low risk” score, (2) Moderate; for studies that at least one of their domains was given “unclear risk” score, (3) High; for studies that at least one of their domains was given “high risk”. Two independent researchers separately assessed the risk of bias for included studies.

### 2.5 Statistical analysis

The overall effect sizes were computed through the use of mean changes and their SDs of sarcopenia components in groups of probiotic supplementation and control. In cases where mean changes were not reported, changes in values of sarcopenia components during the intervention were considered to compute the mean changes. Furthermore, the method of Hozo et al (Hozo et al., 2005) was used for the conversion of standard errors (SEs), interquartile ranges (IQRs), and 95% confidence intervals (CIs) to SDs. The random effects model assumes that the true effect could vary from study to study due to the differences (heterogeneity) among studies but the fixed effect model assumes one true effect size underlies all the studies in the meta‐analysis, so on the random‐effects model which takes between‐study variations into account was used to obtain the overall effect sizes. In addition, *I* ^2^ statistic and Cochrane's *Q* test were used to examine heterogeneity. *I* ^2^ value >50% or *p* < .05 for the *Q*‐test was considered as significant between‐study heterogeneity. Subgroup analysis was conducted based on categorical confounders such as duration of the intervention (≤12 or >12 weeks), to explore the probable source of heterogeneity. Additionally, the included studies have reported the muscle mass in two different scales (kg and percentage); so, subgroup analysis was performed for this variable based on scale (kg and percentage). The extent to which inferences might depend on a particular study was assessed through the use of sensitivity analysis. Visual inspection of Begg's funnel plots and statistical assessment of its funnel plot asymmetry by Begg's test and Egger's test were used to assess publication bias. The STATA version 14.0 (STATA Crop, college station, TX, USA) was used for statistical analyses. *p* values <.05 were considered statistically significant for all tests including Cochran's *Q* test.

## 3 RESULTS

### 3.1 Study characteristics

**FIGURE 1:**
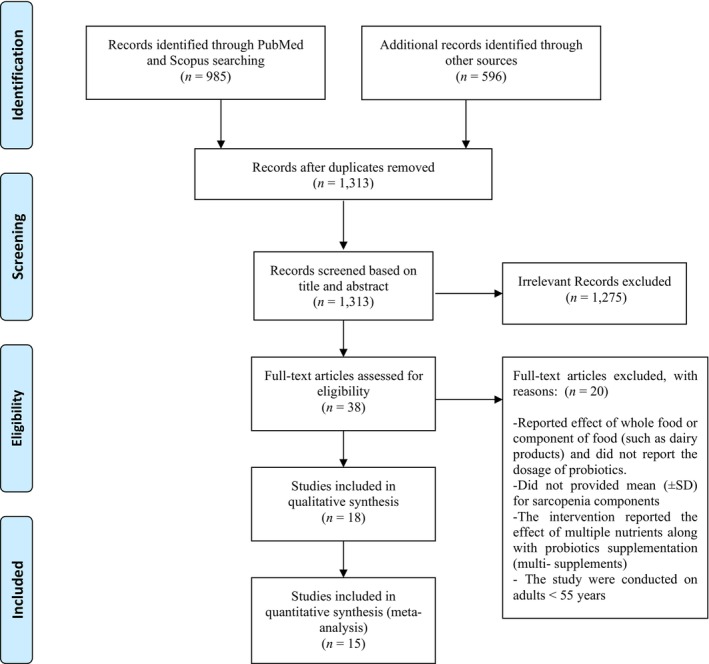
PRISMA flowchart of study selection process for the impact of probiotic supplements on sarcopenia components in elderly adults.

Figure 1 depicts the flowchart of study selection. From different database searching, 3371 articles were detected. After removing duplicate records, 1352 studies were remained for screening. Based on title and abstract screening, 1275 records were excluded and the full text of 42 studied were reviewed more precisely. Twenty studies were excluded with reasons (Supplementary File 2), and then 22 studies were included in the systematic review (Table 1). Regarding meta‐analysis, three studies were not eligible to enter the analysis. Therefore, only 17 studies were recruited for the meta‐analysis (Borzabadi et al., 2018; Ford et al., 2020; Hwang et al., 2019; Inoue et al., 2018; Karim et al., 2022; Lei et al., 2016; Lopes et al., 2018; Neto et al., 2013; Nilsson et al., 2018; Román et al., 2019; Sato et al., 2017; Shinkai et al., 2013; Skrypnik et al., 2019; Szulińska et al., 2018; Tamtaji et al., 2019; Yamamoto et al., 2019) due to following explanations: First, there was only one study that evaluated the effects of probiotic supplementation on waist‐to‐hip ratio (WHR). The results of this study showed a significant reduction in WHR in both probiotic and placebo groups (Hlivak et al., 2005). Second, Mane et al. did not report the endpoint values of BMI. However, it was declared that no significant change was detected in BMI in either probiotic or placebo group (Mañé et al., 2011). Third, Macfarlane et al. only reported data on weight and had no other anthropometric measurements. No significant difference was reported regarding weight in this study (Macfarlane et al., 2013).

**TABLE 1:** Characteristics of included studies in the systematic review.

Author, year	Sex (F/M), subjects (intervention/placebo)	Country	Age (year)	Type of intervention and dosage (CFU/day)	Type of placebo	Duration (weeks)	Health status of subjects	Measurements
*Muscle mass*								
Inoue et al. (2018))	Female/Male (20/18)	Japan	70.3	Bifidobacterium/1.25 × 10^10^	Water	12	Healthy	Lean body mass (kg) was measured by BIA
Neto et al. (2013)	Female/Male (9/8)	Brazil	67.9	Fructooligosaccharides‐probiotic mixture/1 × 10^9^ probiotic	Maltodextrin	12	Frailty	FFM (kg) was measured by BIA
Nilsson et al. (2018)	Female (45/45)	Sweden	76	*Lactobacillus reuteri* 6475/1 × 10^10^	Maltodextrin	46	≤1 SD for BMD	Lean body mass (kg) was measured by BIA
Skrypnik et al. (2019)	Female (26/24)	Poland	56	Probiotic mixture/2.5 × 10^9^	Placebo	12	Obesity (BMI > 30 kg/m^2^)	FFM% was measured by BIA
Skrypnik et al. (2019)	Female (23/24)	Poland	56	Probiotic mixture/1 × 10^10^	Placebo	12	Obesity (BMI > 30 kg/m^2^)	FFM% was measured by BIA
Szulińska et al. (2018)	Female (23/24)	Poland	56	Probiotic mixture/1 × 10^10^	Placebo	12	Obesity (BMI = 30–45 kg/m^2^)	FFM% was measured by BIA
Szulińska et al. (2018)	Female (24/24)	Poland	56	Probiotic mixture/2.5 × 10^9^	Placebo	12	Obesity (BMI = 30‐45 kg/m^2^)	FFM% was measured by BIA
Ford et al. (2020)	Female (7/6)	USA	73.7	HPD plus multistrain probiotic/1.54 × 10^9^	Encapsulated potato starch	18	Healthy	FFM% was measured by BIA
Karim et al. (2022)	Male (47/53)	Pakistan	68	Probiotic mixture/112 billion live bacteria per 1 capsule	Maltose, anticaking agent: silicon dioxide	16	Chronic obstructive pulmonary disease (COPD) patients	Appendicular skeletal mass (kg) by BIA
Karim et al. (2022)	Male (44/48)	Pakistan	67	Probiotic mixture/112 billion live bacteria per 1 capsule	Maltose, anticaking agent: silicon dioxide	12	Patients with chronic heart failure (CHF).	Appendicular skeletal mass (kg) by BIA
*Muscle strength*								
Lei et al. (2016)	Female/Male (189/192)	China	>60	Lactobacillus/6 × 10^9^	Placebo	24	Nondisplaced distal radius fracture	Hand grip strength
Neto et al. (2013)	Female/Male (9/8)	Brazil	67.9	Fructooligosaccharides‐probiotic mixture/1 × 10^9^ probiotic	Maltodextrin	12	Frailty	Hand grip strength
Ford et al. (2020)	Female (21/23)	USA	73.7	HPD plus multistrain probiotic/1.54 × 10^9^	Encapsulated potato‐starch	18	Healthy	Hand grip strength
Román et al. (2019)	Female/Male (17/18)	Spain	65.8	Probiotic mixture/450 billion live bacteria per 4.4 g sachet	Maltose and silicon dioxide	12	Cognitive dysfunction	Hand grip strength
Karim et al. (2022)	Male (47/53)	Pakistan	68	Probiotic mixture/112 billion live bacteria per 1 capsule	Maltose, anticaking agent: silicon dioxide	16	Chronic obstructive pulmonary disease (COPD) patients	Handgrip strength
Karim et al. (2022)	Male (44/48)	Pakistan	67	Probiotic mixture/112 billion live bacteria per 1 capsule	Maltose, anticaking agent: silicon dioxide	12	Patients with chronic heart failure (CHF).	Handgrip strength
*Muscle function*								
Román et al. (2019)	Female/Male (17/18)	Spain	65.8	Probiotic mixture/450 billion live bacteria per 4.4 g sachet	Maltose and silicon dioxide	12	Cognitive dysfunction	Gait speed
Karim et al. (2022)	Male (47/53)	Pakistan	68	Probiotic mixture/112 billion live bacteria per 1 capsule	Maltose, anticaking agent: silicon dioxide	16	Chronic obstructive pulmonary disease (COPD) patients	Gait speed
Karim et al. (2022)	Male (44/48)	Pakistan	67	Probiotic mixture/112 billion live bacteria per 1 capsule	Maltose, anticaking agent: silicon dioxide	12	Patients with chronic heart failure (CHF).	Gait speed
*Body mass index*								
Inoue et al. (2018)	Female/Male (20/18)	Japan	71	Bifidobacterium/1.25 × 10^10^	Water	12	Healthy	Body mass index (kg/m^2^)
Hwang et al. (2019)	Female/Male (45/47)	South Korea	68.0	*Lactobacillus plantarum* C29/1.25 × 10^10^	Cellulose	12	Mild Cognitive Impairment	Body mass index (kg/m^2^)
Mañé et al. (2011)	Female/Male (19/18)	Spain	71	Probiotic mixture/5.10^10^	Placebo	12	Healthy	Body mass index (kg/m^2^)
Mañé et al. (2011)	Female/Male (13/18)	Spain	70	Probiotic mixture/5.10^8^	Placebo	12	Healthy	Body mass index (kg/m^2^)
Sato et al. (2017)	Female/Male (34/34)	Japan	65	Lactobacillus‐fermented milk/4 × 10^10^	Placebo	16	Type 2 diabetes	Body mass index (kg/m^2^)
Shinkai et al. (2013)	Female/Male (92/93)	Japan	70·9	*Lactobacillus pentosus* strain b240/2 × 10^9^	Placebo	20	Healthy	Body mass index (kg/m^2^)
Shinkai et al. (2013)	Female/Male (93/93)	Japan	70·9	*Lactobacillus pentosus* strain b240/2 × 10^10^	Placebo	20	Healthy	Body mass index (kg/m^2^)
Szulińska et al. (2018))	Female (23/24)	Poland	56	Probiotic mixture/1 × 10^10^	Placebo	12	Obesity	Body mass index (kg/m^2^)
Szulińska et al. (2018)	Female (24/24)	Poland	56	Probiotic mixture/2.5 × 10^9^	Placebo	12	Obesity	Body mass index (kg/m^2^)
Lopes et al. (2018)	Female/Male (29/29)	Brazil	63.1	Probiotic dairy drink/7.4 × 10^8^ ± 5.4 × 10^8^ CFU/100 mL	Milk	7	CKD	Body mass index (kg/m^2^)
Yamamoto et al. (2019)	Female/Male (44/52)	Japan	88.3	Yogurt enriched with mixed probiotics/1.8 and 3.5 × 10^8^	Yogurt	12	Healthy	Body mass index (kg/m^2^)
Neto et al. (2013)	Female/Male (9/8)	Brazil	67.9	Fructooligosaccharides‐probiotic mixture/1 × 10^9^probiotic	Maltodextrin	12	Frailty	Body mass index (kg/m^2^)
Tamtaji et al. (2019)	Female/Male (27/26)	Iran	76.2	Mixed probiotic /2 × 10^9^	Starch	12	Alzheimer's disease	Body mass index (kg/m^2^)
Borzabadi et al. (2018)	Female/Male (25/25)	Iran	66.8	Probiotic mixture/8 × 10^9^	Placebo	12	Parkinson's disease	Body mass index (kg/m^2^)
*Body weight*								
Hwang et al. (2019)	Female/Male (45/47)	South Korea	68.0	*Lactobacillus plantarum* C29/1.25 × 10^10^	Cellulose	12	Mild Cognitive Impairment	Body weight (kg)
Yamamoto et al. (2019)	Female/Male (44/52)	Japan	88.3	Yogurt enriched with mixed probiotic/1.8 and 3.5× 10^8^	Yogurt	12	Healthy	Body weight (kg)
Tamtaji et al. (2019)	Female/Male (27/26)	Iran	76.2	Probiotic mixture/2 × 10^9^	Starch	12	Alzheimer's disease	Body weight (kg)
Borzabadi et al. (2018)	Female/Male (25/25)	Iran	66.8	Probiotic mixture/8 × 10^9^	Placebo	12	Parkinson's disease	Body weight (kg)
Macfarlane et al. (2013)	Female/Male (23/20)	UK	71.9	B. longum/2 × 10^11^	Potato and maltodextrin	4	Healthy	Weight(kg)
*Body mass*								
Inoue et al. (2018)	Female/Male (20/18)	Japan	71	Bifidobacterium/1.25 × 10^10^	Water	12	Healthy	Body mass (kg)
Skrypnik et al. (2019)	Female (23/24)	Poland	56	Probiotic mixture/1 × 10^10^	Placebo	12	Obesity	Body mass (kg)
Skrypnik et al., 2019	Female (26/24)	Poland	56	Probiotic mixture/2.5 × 10^9^	Placebo	12	Obesity	Body mass (kg)
Szulińska et al. (2018)	Female (23/24)	Poland	56	Probiotic mixture/1 × 10^10^	Placebo	12	Obesity	Body mass (kg)
Szulińska et al. (2018)	Female (24/24)	Poland	56	Probiotic mixture/2.5 × 10^9^	Placebo	12	Obesity	Body mass (kg)
Neto et al. (2013)	Female/Male (9/8)	Brazil	67.9	Probiotic mixture with Fructooligosaccharides/1 × 10^9^ probiotic	Maltodextrin	12	Frailty	Body mass (kg)
*Waist circumference*								
Szulińska et al. (2018)	Female (23/24)	Poland	56	Probiotic mixture/1 × 10^10^	Placebo	12	Obesity	Waist circumference (cm)
Szulińska et al. (2018)	Female (24/24)	Poland	56	Probiotic mixture/2.5 × 10^9^	Placebo	12	Obesity	Waist circumference (cm)
*Waist‐to‐hip ratio*								
Hlivak et al. (2005)	Female/Male (20/18)	Slovakia	75.35	probiotic mixture (EF M‐74)/2 × 10^9^	Placebo	12	Healthy	Waist to hip ratio

Abbreviations: BIA, bioelectrical impedance analysis; BMD, bone mineral density; BMI, body mass index; CFU, colony‐forming units; CKD, chronic kidney disease; cm, centimeters; F, female; FFM, fat‐free mass; kg, kilogram; M, male; SD, standard deviation.

Totally, 1589 participants were included in the systematic review (adults more than 55 years). Six studies enrolled healthy participants (Ford et al., 2020; Hlivak et al., 2005; Inoue et al., 2018; Macfarlane et al., 2013; Mañé et al., 2011; Shinkai et al., 2013), and other studies recruited people with comorbidities, such as obesity, metabolic syndrome, sarcopenia or frailty, chronic obstructive pulmonary disease (COPD), chronic heart failure, and nervous system. Seven studies had duration more than 12 weeks (Ford et al., 2020; Karim et al., 2022; Lei et al., 2016; Nilsson et al., 2018; Sato et al., 2017; Shinkai et al., 2013) and other studies had duration equal and less than 12 weeks. In five records, foods were enriched by probiotics with certain dosage and applied for intervention group (Ford et al., 2020; Hwang et al., 2019; Lopes et al., 2018; Sato et al., 2017; Yamamoto et al., 2019), and probiotic supplements were used in 15 remaining studies. The main strain type of probiotic supplements was Lactobacillus and Bifidobacteria. Most studies administered supplement as probiotic mixtures (Borzabadi et al., 2018; Ford et al., 2020; Karim et al., 2022; Mañé et al., 2011; Román et al., 2019; Skrypnik et al., 2019; Szulińska et al., 2018; Tamtaji et al., 2019; Yamamoto et al., 2019), and one of them used probiotic supplement enriched with oligosaccharides (Neto et al., 2013). The nature of placebo in the included studies was water, maltose, maltodextrin, cellulose, and potato.

### 3.2 Risk of bias assessment

Based on the criteria of the applied tool designed for randomized clinical trials, the Cochrane quality assessment tool, six studies that had a low risk of bias (Ford et al., 2020; Inoue et al., 2018; Lei et al., 2016; Nilsson et al., 2018; Román et al., 2019; Skrypnik et al., 2019); (Borzabadi et al., 2018; Hlivak et al., 2005; Hwang et al., 2019; Karim et al., 2022; Lopes et al., 2018; Macfarlane et al., 2013; Sato et al., 2017; Shinkai et al., 2013; Tamtaji et al., 2019; Yamamoto et al., 2019) (Supplementary File 3). Two studies conducted by Valentini Neto et al. and Ford et al. had a moderate risk of bias (Ford et al., 2020; Neto et al., 2013), and the overall risk of bias in others was defined as high.

### 3.3 The impact of probiotic supplements on fat‐free mass and muscle mass

**FIGURE 2:**
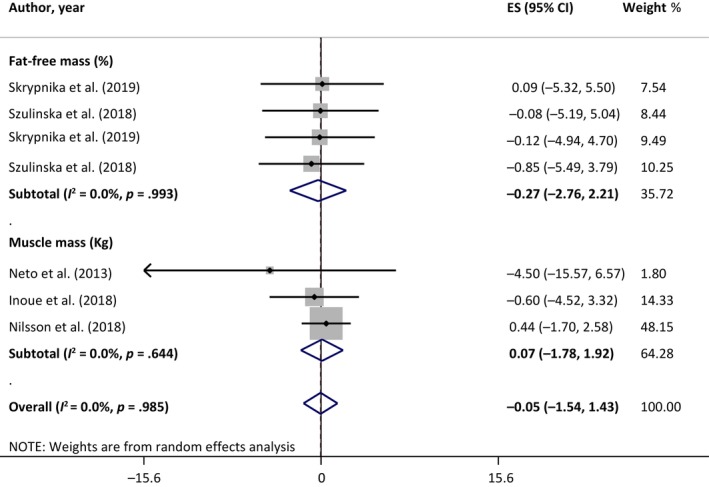
Forest plot of the effect of probiotic supplementation on muscle mass.

The overall effect of meta‐analysis on seven studies showed a positive effect of probiotic supplementation on muscle mass (WMD: 0.50, 95% CI: 0.01, 0.99; *I* ^2^: 0.0%, *p* = .997). Moreover, subgroup analysis based on measurement methods, including appendicular muscle mass (kg) and fat‐free mass (%), revealed significant beneficial effect of probiotic supplementation on muscle mass (kg) (WMD: 0.55, 95% CI: 0.05, 1.05; *I* ^2^: 0.0%, *p* = .995). No significant effect of probiotic supplementation on fat‐free mass (%) was detected (WMD: −0.50, 95% CI: −2.76, 1.76; *I* ^2^: 0.0%, *p* = .992) (Figure 2).

### 3.4 The impact of probiotic supplements on muscle strength

**FIGURE 3:**
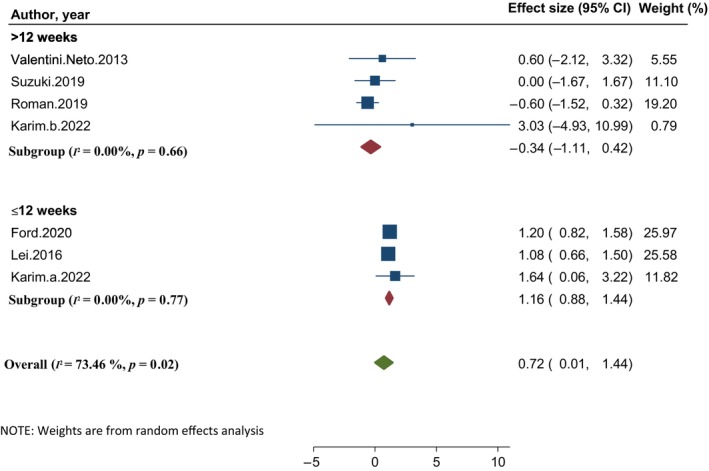
Forest plot of the effect of probiotic supplementation on muscle strength.

Based on the pooled overall effect of seven studies with total of 701 participants, probiotic supplementation had a significant effect on muscle strength (WMD: 0.72, 95% CI: 0.1, 1.44; *I* ^2^: 73.46%, *p* = .02) (Figure 3). To reduce the substantial heterogeneity, a subgroup analysis was run based on the duration of interventions. The result of the analysis showed that probiotic supplementation for more than 12 weeks significantly increased muscle strength (WMD: 1.16, 95% CI: 0.88, 1.44; *I* ^2^: 0.0%, *p* = .77). However, probiotic supplementation of less than 12 weeks had no effect on muscle strength (WMD: −0.34, 95% CI: −1.11, 0.42; *I* ^2^: 0.0%, *p* = .66) (Figure 3).

### 3.5 The impact of probiotic supplements on muscle function

**FIGURE 4:**
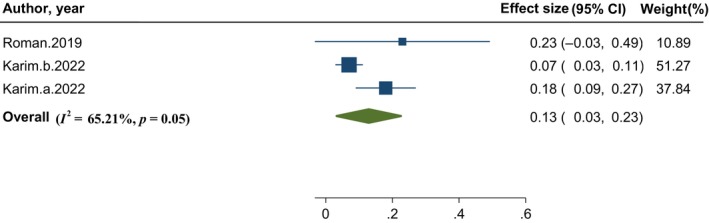
Forest plot of the effect of probiotic supplementation on muscle function.

The result of pooled analysis showed a significant effect of probiotic supplementation on the improvement of muscle function (WMD: 0.13, 95% CI: 0.03, 0.23; *I* ^2^: 65.6%, *p* = .05). The source of relatively high heterogeneity was not detectable due to limited number of studies **(**Figure 4).

### 3.6 The impact of probiotic supplements on anthropometric measurements

The results of meta‐analysis on anthropometric measurements including body mass index and body mass are presented in Table 2. Accordingly, probiotic supplementation had no significant effect on anthropometric indices, including body mass (SMD: −0.05, 95% CI: −2.57, 1.56; *I* ^2^: 0.0%, *p* = .976) and body mass index (SMD: 0.08, 95% CI: −0.16, 0.32; *I* ^2^: 0.0%, *p* = .718). In addition, there was no substantial heterogeneity between studies in all analyses.

**TABLE 2:** Effects of probiotic supplementation on anthropometric measurements.

	No. of studies	SMD (95%CI)	*I* ^2^ (%)	*p*‐value for heterogeneity
Body mass index	12	0.08 (−0.16, 0.32)	0.0	.718
Body mass	6	−0.05 (−2.57, 1.56)	0.0	.976

### 3.7 Sensitivity analysis and publication bias

According to the sensitivity analysis, there was no significant effect of each single study on pooled effect sizes of fat‐free mass, muscle strength (≤12 weeks and >12 weeks), muscle function, body mass, and BMI. However, sign of publication bias for studies investigating the effect of probiotics on muscle mass assessed by the Begg et al statistical tests was observed (*p* = .0275). By removing Karim et al. studies, the overall effect of probiotic supplementation did not remain significant (WMD: 0.57, 95% CI: −0.048, 1.19 (Karim et al., 2022) and WMD: 0.48, 95% CI: −0.27, 1.24 (Karim et al., 2022)). There were no evidences of publication bias for studies examining the effect of probiotics on muscle mass, muscle strength and muscle function, body mass, BMI, and body weight.

## 4 DISCUSSION

The main results of this systematic review and meta‐analysis point out a positive effect of probiotic supplementation on muscle mass and muscle function. In addition, findings of subgroup analysis affirmed that probiotic supplementation for more than 12 weeks can improve muscle strength significantly.

Recently, the important role of gut microbiota in inducing age‐related muscle dysfunction has been suggested (Chen et al., 2022; Ticinesi et al., 2017). Consistent with the findings of this study, animal research showed that probiotic/prebiotic administration could ameliorate age‐related muscle dysfunctions and improve Sarcopenia features through the gut–muscle axis (Chen et al., 2022; Liu et al., 2021). The abnormal gut microbiota species and numbers can occur simultaneously as a consequence of aging (Ni Lochlainn et al., 2018). It is shown that the alterations of normal fecal microbiota composition could result in a high pro‐inflammatory micro‐environment (Bjørkhaug et al., 2019). In this regard, the results of a systematic review and meta‐analysis showed that the elevated systematic inflammation across the lifespan was associated with Sarcopenia among older adults (Shokri‐Mashhadi et al., 2021). Interestingly, the results of a previous systematic review and meta‐analysis confirmed that probiotic supplementation has beneficial effects on circulating inflammatory biomarkers in health and disease conditions (Kazemi et al., 2020). Although the underlying causes of chronic inflammation are varied, it is supposed that changes in gut microbiota have a crucial role in the pathogenesis of this disorder (Ferrucci & Fabbri, 2018; Ticinesi et al., 2017). In addition, it is believed that higher inflammation is an important risk factor for muscle atrophy and malfunction through interfering with muscle anabolism and energy homeostasis (Belizário et al., 2016). Thus, it is likely that probiotic supplementation improves muscle health and function through inflammation reduction.

Reduced levels of IGF‐1 (Insulin‐like growth factor 1) simultaneously during a lifetime may be another explanation for the potential association between probiotics and sarcopenia (Barbieri et al., 2003). In this regard, lower handgrip strength and worse physical performance are documented in elderly persons with lower levels of IGF‐1 levels (Hor et al., 2021; Van Nieuwpoort et al., 2018). Surprisingly, the predictive role of IGF‐1 levels on muscle function is only observed in participants with the lowest levels of inflammation (IL‐6 levels); explaining the mediatory role of inflammation between IGF‐1 and muscle strength and function (Barbieri et al., 2003). It is previously well indicated that low expression of IGF‐1 is correlated with impaired differentiation of myotubes resulting in reduced size and dysfunction of skeletal muscles (Hor et al., 2021). Moreover, animal studies suggested that *Bifidobacterium Infantis* administration may upregulate IGF‐1 expression subsequent to lipopolysaccharide injection (Wang et al., 2019). Given the mentioned reasons, the gut–muscle axis might clarify how probiotic supplementation can affect gut microorganism composition and increase overall health resulting from the improvement of inflammation and immune system function in older adults (Sanchez et al., 2017).

In addition to the improvement in muscle strength, some preceding research proposed the possible favorable effect of probiotics on muscle mass (Ticinesi et al., 2019). Previous investigations in animal models demonstrated that alteration of gut microbiota can positively affect skeletal muscle mass and function (Lahiri et al., 2019). Also, the administration of probiotics in mouse models with muscle disorder improved muscle mass (Chen et al., 2022; Varian et al., 2016). A randomized clinical trial demonstrated that using the probiotic can increase the lean body mass and skeletal muscle mass in long‐distance runners (Smarkusz‐Zarzecka et al., 2020). In addition, taking the multistrain probiotic improved muscle strength and functional performance in chronic obstructive pulmonary disease (COPD) patients (Karim et al., 2022). A recent review also declared that changes in gut microbiota can affect muscle phenotypes and using probiotics and prebiotics, are potential factors to enhance muscle mass. In this regard, Lactobacillus and Bifidobacterium strains were beneficial in age‐related muscle loss restoring (Liu et al., 2021). In this concern, observational studies indicated that the reduction of gut microbiota biodiversity induced by aging is associated with loss of skeletal muscle and calf circumference reduction (Ren et al., 2021). Additionally, the association between body mass index (BMI) and microbiota composition has been reported in Ukrainian obese persons (Koliada et al., 2017).

Clinically, based on the last definition provided by the European Working Group on age‐related sarcopenia, the reliability of muscle strength in predicting sarcopenia and its adverse outcomes is even more than muscle mass (Cruz‐Jentoft et al., 2019). The outcomes of the present study emphasize the clinical prominence of probiotic supplementation on the most reliable diagnostic sarcopenia component, muscle strength. Moreover, the outcomes of this study may make known new insight regarding the important role of gastrointestinal tract on muscle function and dysfunction, and the assessment of the intestinal microbiota diversity can be a good prognostic tool for impaired muscle function. Furthermore, appropriate subgroup analysis enabled us to elicit a remarkable association between probiotic supplementation and muscle strength. These findings have recommended that the long‐time application of probiotics could be also justified for the prevention of age‐related muscle dysfunction. However, some limitations must be considered. First, all studies with various comorbidities from a range of metabolic dysfunction and inpatients population to obese participants were included in the analysis that might influence the results. Since the underlying mechanism of sarcopenia might be different in sarcopenic‐obese participants rather than in elderly ones. Nevertheless, the limited number of studies that have been done among obese participants, consequently, subgroup analysis was not applicable. In addition, the available health status diversity (healthy vs. ill) between included studies might disturb the final findings. Second, due to a lack of evidence, it was not possible to perform analysis on inflammatory markers as well as IGF‐1 levels to reinforce the proposed mechanism in the present study. The identifying possible mechanism underlying the positive effect of probiotics administration on muscle strength, mass, and function in elderly participants may recommend the specific probiotic preparation for a specific population group. So, more clinical trials on sarcopenic older participants with simultaneous assessment inflammation as well as other possible pathogenesis variables, including adipokines levels, oxidative stress markers, and insulin resistance, should be taken into concern for (Polyzos & Margioris, 2018) possible mechanism underlying this finding.

## 5 CONCLUSION

In conclusion, this study declared the positive impact of probiotic supplementation on the most reliable diagnostic sarcopenia component, muscle strength particularly in more than 12 weeks' application as well as muscle mass and function. However, more clinical trials on sarcopenic older participants with simultaneous assessment of inflammation as well as other possible pathogenesis variables are recommended.

## AUTHOR CONTRIBUTIONS

**Nafiseh Shokri‐Mashhadi:** Conceptualization (equal); methodology (lead); supervision (lead); writing – original draft (equal); writing – review and editing (equal). **Fatemeh Navab:** Data curation (equal); investigation (equal). **Shakila Ansari:** Writing – review and editing (equal). **Mohammad Hossein Rouhani:** Formal analysis (lead). **Zahra Hajhashemy:** Data curation (equal); investigation (equal). **Sahar Saraf‐Bank:** Conceptualization (equal); writing – original draft (equal).

## FUNDING INFORMATION

This study was supported by Isfahan University of Medical Science. The funders had no role in the study design, data collection, and analysis, decision to publish, or preparation of the manuscript.

## CONFLICT OF INTEREST STATEMENT

The authors declare that they have no conflict of interest.

## ETHICAL APPROVAL AND CONSENT TO PARTICIPATE

This study does not involve any human or animal testing. This study protocol was reviewed and approved by the Ethic committee of Isfahan University of Medical Science, IR.MUI.RESEARCH.REC.1399.324.

## Supporting information


Supplementary File 1

Supplementary File 2

Supplementary File 3

## Data Availability

All data generated or analyzed during this study are included in this article and its supplementary material files. Further inquiries can be directed to the corresponding author.
