# Supplementary material for: A meta‐analysis of the effect of probiotic administration on age‐related sarcopenia
Source: Food Sci Nutr. 2023 Aug 9;11(9):4975–87. doi: 10.1002/fsn3.3515 (PMC10494607; doi:10.1002/fsn3.3515)
Supplement: Supplementary file 1 [file FSN3-11-4975-s003.docx]

| **Supplementary file 1**. Search Results.  PubMed search results *(end of juli.2021)* | |
| --- | --- |
| **#1** (((((Sarcopenia[MeSH Terms]) OR (Muscle strength[Title/Abstract])) OR (saropenia[Title/Abstract])) OR ("Hand strength"[Title/Abstract])) OR ("Physical performance"[Title/Abstract])) OR ("Muscle function*"[Title/Abstract]) OR (Frailty[Title/Abstract]) OR ("Walking Speed"[Title/Abstract]) OR ("Gait speed"[Title/Abstract]) OR ("Grip strength*"[Title/Abstract]) OR ("Hand grip*"[Title/Abstract]) OR ("lean body mass"[Title/Abstract]) OR ("Percentage of body fat"[Title/Abstract]) OR ("Knee extension strength*"[Title/Abstract]) | 593,183 |
| **#2** ((((((((((Probiotic*[Title/Abstract]) OR (probiotic*[MeSH Terms])) OR ("Escherichia coli"[MeSH Terms])) OR (Microbiota*[Title/Abstract])) OR (Bifidobacterium[Title/Abstract])) OR (Lactobacillus[Title/Abstract])) OR (Saccharomyces[Title/Abstract])) OR (kefir[Title/Abstract])) OR (Yogurt[Title/Abstract])) OR ("Escherichia coli"[Title/Abstract])) | 97,763 |
| #1 AND #2 | 306 |

| Scopus search results *(end of juli.2021)* | |
| --- | --- |
| **#1** TITLE-ABS ( sarcopenia  OR  "Muscle strength"  OR  "Hand strength"  OR  "Physical performance"  OR  "Muscle function*"  OR  sarcopenia  OR  frailty  OR  "Walking Speed"  OR  "Gait speed"  OR  "Grip strength*"  OR  "Hand grip*"  OR  "lean body mass"  OR  "Percentage of body fat"  OR  "Knee extension strength) | 210,219 |
| **#2** TITLE-ABS (Probiotic* OR “Escherichia coli” OR Microbiota* OR Bifidobacterium OR Lactobacillus OR Saccharomyces OR kefir OR Yogurt) | 880,716 |
| #1 AND #2 | 679 |

| Web of Science search results *(end of juli.2021)* | |
| --- | --- |
| **#1** TOPIC: (sarcopenia  OR  "Muscle strength"  OR  "Hand strength"  OR  "Physical performance"  OR  "Muscle function*"  OR  sarcopenia  OR  frailty  OR  "Walking Speed"  OR  "Gait speed"  OR  "Grip strength*"  OR  "Hand grip*"  OR  "lean body mass"  OR  "Percentage of body fat"  OR  "Knee extension strength*") | 129,174 |
| **#2** TOPIC: (Probiotic* OR “Escherichia coli” OR Microbiota* OR Bifidobacterium OR Lactobacillus OR Saccharomyces OR kefir OR Yogurt) | 751,908 |
| #1 AND #2 | 447 |

| **Cochrane library** *(end of juli.2021)* | |
| --- | --- |
| **#1** TITLE-ABS-KEY (sarcopenia  OR  "Muscle strength"  OR  "Hand strength"  OR  "Physical performance"  OR  "Muscle function*"  OR  sarcopenia  OR  frailty  OR  "Walking Speed"  OR  "Gait speed"  OR  "Grip strength*"  OR  "Hand grip*"  OR  "lean body mass"  OR  "Percentage of body fat"  OR  "Knee extension strength*") | 34,034 |
| **#2** TITLE-ABS-KEY (Probiotic* OR “Escherichia coli” OR Microbiota* OR Bifidobacterium OR Lactobacillus OR Saccharomyces OR kefir OR Yogurt) | 17,055 |
| #1 AND #2 | 149 |
