# Supplementary material for: A meta‐analysis of the effect of probiotic administration on age‐related sarcopenia
Source: Food Sci Nutr. 2023 Aug 9;11(9):4975–87. doi: 10.1002/fsn3.3515 (PMC10494607; doi:10.1002/fsn3.3515)
Supplement: Supplementary file 2 [file FSN3-11-4975-s002.docx]

**Supplementary file 2:** List of excluded studies after full-text assessment

| **Reasons for exclusion** | **Reference** |
| --- | --- |
| Reported effect of fermented whole food (such as dairy products) and did not report the dosage or strain of probiotics | (1-7) |
| did not report the predefined endpoints as mean (±SD) for sarcopenia components | (8-15) |
| The intervention reported the effect of multiple nutrients along with probiotics supplementation (multi- supplements) | (2, 16) |
| The study were conducted on adults(< 55 years) | (17-20) |

1. Aoyagi Y, Amamoto R, Park S, Honda Y, Shimamoto K, Kushiro A, et al. Independent and interactive effects of habitually ingesting fermented milk products containing Lactobacillus casei strain Shirota and of engaging in moderate habitual daily physical activity on the intestinal health of older people. Frontiers in microbiology. 2019;10:1477.

2. Kleessen B, Sykura B, Zunft H-J, Blaut M. Effects of inulin and lactose on fecal microflora, microbial activity, and bowel habit in elderly constipated persons. The American journal of clinical nutrition. 1997;65(5):1397-402.

3. Picca A, Ponziani FR, Calvani R, Marini F, Biancolillo A, Coelho-Júnior HJ, et al. Gut microbial, inflammatory and metabolic signatures in older people with physical frailty and sarcopenia: Results from the BIOSPHERE Study. Nutrients. 2020;12(1):65.

4. Thomas DT, Wideman L, Lovelady CA. Effects of a dairy supplement and resistance training on lean mass and insulin-like growth factor in women. International journal of sport nutrition and exercise metabolism. 2011;21(3):181-8.

5. Vulevic J, Drakoularakou A, Yaqoob P, Tzortzis G, Gibson GR. Modulation of the fecal microflora profile and immune function by a novel trans-galactooligosaccharide mixture (B-GOS) in healthy elderly volunteers. The American journal of clinical nutrition. 2008;88(5):1438-46.

6. Walton GE, van den Heuvel EG, Kosters MH, Rastall RA, Tuohy KM, Gibson GR. A randomised crossover study investigating the effects of galacto-oligosaccharides on the faecal microbiota in men and women over 50 years of age. British Journal of Nutrition. 2012;107(10):1466-75.

7. Alemán-Mateo H, Carreón VR, Macías L, Astiazaran-García H, Gallegos-Aguilar AC, Enríquez JRR. Nutrient-rich dairy proteins improve appendicular skeletal muscle mass and physical performance, and attenuate the loss of muscle strength in older men and women subjects: a single-blind randomized clinical trial. Clinical interventions in aging. 2014;9:1517.

8. Ford AL, Nagulesapillai V, Piano A, Auger J, Girard S-A, Christman M, et al. Microbiota stability and gastrointestinal tolerance in response to a high-protein diet with and without a prebiotic, probiotic, and synbiotic: A randomized, double-blind, placebo-controlled trial in older women. Journal of the Academy of Nutrition and Dietetics. 2020;120(4):500-16. e10.

9. Guillemard E, Tondu F, Lacoin F, Schrezenmeir J. Consumption of a fermented dairy product containing the probiotic Lactobacillus casei DN-114 001 reduces the duration of respiratory infections in the elderly in a randomised controlled trial. British journal of nutrition. 2010;103(1):58-68.

10. Theou O, Jayanama K, Fernández-Garrido J, Buigues C, Pruimboom L, Hoogland A, et al. Can a prebiotic formulation reduce frailty levels in older people? The Journal of frailty & aging. 2019;8(1):48-52.

11. Schiffrin E, Thomas D, Kumar V, Brown C, Hager C, Van't Hof M, et al. Systemic inflammatory markers in older persons: the effect of oral nutritional supplementation with prebiotics. The journal of nutrition, health & aging. 2007;11(6):475.

12. Takimoto T, Hatanaka M, Hoshino T, Takara T, Tanaka K, Shimizu A, et al. Effect of Bacillus subtilis C-3102 on bone mineral density in healthy postmenopausal Japanese women: a randomized, placebo-controlled, double-blind clinical trial. Bioscience of microbiota, food and health. 2018:18-006.

13. Kassaian N, Feizi A, Aminorroaya A, Jafari P, Ebrahimi MT, Amini M. The effects of probiotics and synbiotic supplementation on glucose and insulin metabolism in adults with prediabetes: a double-blind randomized clinical trial. Acta diabetologica. 2018;55(10):1019-28.

14. Ahmed M, Prasad J, Gill H, Stevenson L, Gopal P. Impact of consumption of different levels of Bifidobacterium lactis HN019 on the intestinal microflora of elderly human subjects. Journal of Nutrition Health and Aging. 2007;11(1):26.

15. Akatsu H, Iwabuchi N, Xiao Jz, Matsuyama Z, Kurihara R, Okuda K, et al. Clinical effects of probiotic Bifidobacterium longum BB536 on immune function and intestinal microbiota in elderly patients receiving enteral tube feeding. Journal of Parenteral and Enteral Nutrition. 2013;37(5):631-40.

16. Dos Santos PQ, Guedes JC, de Jesus RP, Dos Santos RR, Fiaconne RL. Effects of using symbiotics in the clinical nutritional evolution of patients with chronic pancreatitis: Study prospective, randomized, controlled, double blind. Clinical nutrition ESPEN. 2017;18:9-15.

17. Carbuhn AF, Reynolds SM, Campbell CW, Bradford LA, Deckert JA, Kreutzer A, et al. Effects of Probiotic (Bifidobacterium longum 35624) Supplementation on Exercise Performance, Immune Modulation, and Cognitive Outlook in Division I Female Swimmers. Sports (Basel, Switzerland). 2018;6(4).

18. Cox AJ, Pyne DB, Saunders PU, Fricker PA. Oral administration of the probiotic Lactobacillus fermentum VRI-003 and mucosal immunity in endurance athletes. British journal of sports medicine. 2010;44(4):222-6.

19. Shing CM, Peake JM, Lim CL, Briskey D, Walsh NP, Fortes MB, et al. Effects of probiotics supplementation on gastrointestinal permeability, inflammation and exercise performance in the heat. European Journal of Applied Physiology. 2014;114(1):93-103.

20. Gill SK, Allerton DM, Ansley-Robson P, Hemmings K, Cox M, Costa RJS. Does Short-Term High Dose Probiotic Supplementation Containing Lactobacillus casei Attenuate Exertional-Heat Stress Induced Endotoxaemia and Cytokinaemia? International Journal of Sport Nutrition and Exercise Metabolism. 2016;26(3):268-75.
