# Supplementary material for: A meta‐analysis of the effect of probiotic administration on age‐related sarcopenia
Source: Food Sci Nutr. 2023 Aug 9;11(9):4975–87. doi: 10.1002/fsn3.3515 (PMC10494607; doi:10.1002/fsn3.3515)
Supplement: Supplementary file 3 [file FSN3-11-4975-s001.docx]

| **Supplementary file 3.** The quality assessment of included studies | Random sequence generation (selection bias) | Allocation concealment (selection bias) | Selective reporting (reporting bias) | Other source of bias (detection bias) | Blinding of participants and personnel (performance bias) | Blinding of outcome assessment (detection bias) | Incomplete outcome data addressed (attrition bias) |
| --- | --- | --- | --- | --- | --- | --- | --- |
| Karim, 2022 |  |  |  |  |  |  |  |
| Karim, 2022 |  |  |  |  |  |  |  |
| Ford, 2020 |  |  |  |  |  |  |  |
| Suzuki, 2019 |  |  |  |  |  |  |  |
| Hwang, 2019 |  |  |  |  |  |  |  |
| Lopes, 2019 |  |  |  |  |  |  |  |
| Román, 2019 |  |  |  |  |  |  |  |
| Skrypnik, 2019 |  |  |  |  |  |  |  |
| Yamamoto, 2019 |  |  |  |  |  |  |  |
| Borzabadi, 2019 |  |  |  |  |  |  |  |
| Inoue, 2018 |  |  |  |  |  |  |  |
| Nilsson, 2018 |  |  |  |  |  |  |  |
| Tamtaji, 2018 |  |  |  |  |  |  |  |
| Szulinska, 2018 |  |  |  |  |  |  |  |
| Sato, 2017 |  |  |  |  |  |  |  |
| Lei , 2016 |  |  |  |  |  |  |  |
| Valentini Neto, 2013 |  |  |  |  |  |  |  |
| Macfarlane, 2013 |  |  |  |  |  |  |  |
| Shinkai, 2012 |  |  |  |  |  |  |  |
| Mane, 2011 |  |  |  |  |  |  |  |
| Hlivak, 2005 |  |  |  |  |  |  |  |
| “High risk” (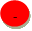), “Low risk” (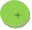) and “Unclear risk” (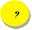) | | | | | | | |
